# Superabsorbent Polymer Network Degradable by a Human Urinary Enzyme

**DOI:** 10.3390/polym13060929

**Published:** 2021-03-17

**Authors:** Minji Whang, Hyeonji Yu, Jungwook Kim

**Affiliations:** Department of Chemical and Biomolecular Engineering, Sogang University, 35 Baekbeom-ro, Mapo-gu, Seoul 04107, Korea; mjwhang@sogang.ac.kr (M.W.); lkjh6309@sogang.ac.kr (H.Y.)

**Keywords:** biodegradable polymer, superabsorbent polymer (SAP), cleavable crosslinker, poly (acrylic acid) (PAA), urokinase-type plasminogen activator (uPA)

## Abstract

Owing to its superior water absorption capacity, superabsorbent polymer (SAP) based on a poly (acrylic acid) network is extensively used in industrial products such as diapers, wound dressing, or surgical pads. However, because SAP does not degrade naturally, a massive amount of non-degradable waste is discarded daily, posing serious environmental problems. Considering that diapers are the most widely used end-product of SAP, we created one that is degradable by a human urinary enzyme. We chose three enzyme candidates, all of which have substrates that were modified with polymerizable groups to be examined for cleavable crosslinkers of SAP. We found that the urokinase-type plasminogen activator (uPA) substrate, end-modified with acrylamide groups at sufficient distances from the enzymatic cleavage site, can be successfully used as a cleavable crosslinker of SAP. The resulting SAP slowly degraded over several days in the aqueous solution containing uPA at a physiological concentration found in human urine and became shapeless in ~30 days.

## 1. Introduction

Superabsorbent polymer, commonly made of acrylic acid-based crosslinked copolymer, absorbs as much as hundreds of times its weight in water due to high osmotic pressure generated by the concentrated charge of the polymer network. Owing to its superior water absorption capacity, fast absorption rate, and low discharge of absorbed water under applied pressure, SAP has been used for various industrial products including diapers, wound dressing, surgical pads, fire-retardant gel, and food additives [1]. The global SAP market was about 2.3 million metric tons in 2020 [2], indicating that a massive amount of SAP waste is discarded daily. Because SAP is naturally non-biodegradable, this waste poses a serious environmental problem, indicating that the development of an eco-friendly and biodegradable SAP is urgent [3]. When developing a biodegradable SAP, a critical consideration is the ability to synthesize SAP using a radical polymerization (the current industrial method) rather than a step polymerization.

Currently, the most commonly used SAP in the industry is a crosslinked network of poly (acrylic acid) (PAA) chains [4]. It has been known that PAA chains of a molecular weight less than 1 kg/mol can be degraded in sewage by soil microorganisms [5]. Therefore, a design principle in creating a biodegradable SAP is first to degrade a crosslinked PAA network into linear PAA chains by cleaving crosslinking sites and then fragmentizing linear PAA chains into smaller pieces to be degraded by the microorganisms. Previous works have synthesized biodegradable SAP through embedding cleavable moieties into the polymer network [6]; however, these attempts had limited success due to complex synthetic routes, low production yield, and insufficient water absorbency and mechanical properties [7,8,9]. Instead of a PAA, other polymers that are charged and intrinsically biodegradable were made into SAPs via crosslinking. For example, a crosslinked network of biodegradable poly (aspartic acid) polymers was synthesized via a condensation polymerization of L-aspartic acid [10]. However, its use in the SAP industry is limited because of its weak mechanical strength and a high production cost. While most previously synthesized biodegradable SAPs contained the ester groups for hydrolytic degradation, these methods cannot apply to the current industrial SAP manufacturing process since it involves a high-temperature heating step, which accelerates hydrolytic degradation. Thus, to realize the industrial application and to enhance the long-term stability during storage, the use of a specific enzyme–substrate reaction for degradation is preferable [11].

**Figure 1:**
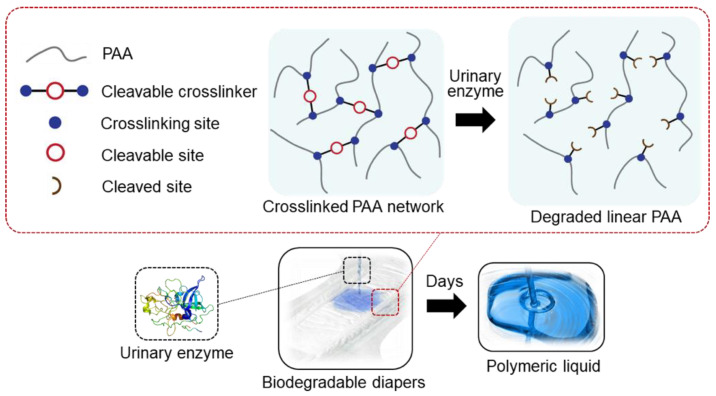
A schematic illustrating the enzymatic degradation of the SAP created using the cleavable crosslinker.

In this study, because diapers are the most widely used end-product of SAP, we created one that is degradable by a human urinary enzyme. If the enzymatic substrates are modified at both ends with polymerizable groups, cleavable crosslinkers that can participate in the radical polymerization of acrylic acid can be created. Because the end modification may sterically hinder the enzymes from binding to the cleavage sites, the enzymatic substrates were examined for their degradability after modification. We created the SAP using the enzymatically cleavable crosslinker and tested the biodegradation of the SAP into linear PAA chains when the enzyme was present in a human urinary concentration (Figure 1). Among various enzymes found in human urine, such as oxidoreductases, transferases, hydrolases, and lyases [12], we selected leucine aminopeptidase (LAP), *β*-glucuronidase (GLU), and urokinase-type plasminogen activator (uPA) (Table S1 in Supplementary Material), all of which are hydrolase and are known to cleave a specific chemical bond.

## 2. Materials and Methods

Leucine aminopeptidase (EC 3.4.1.2) microsomal from porcine kidney (LAP), *β*-glucuronidase (EC 3.2.1.31) from helix pomatia type H-2 (GLU), *N*-(tert-Butoxycarbonyl)glycine, glycine, triglycine, *σ*-pinene, triphosgene, triethylamine, *N*-carboxyanhydride (NCA), 2-aminoethyl methacrylate hydrochloride (2-AMEA), allylamine, 4-nitrophenyl- *β*-D-glucuronide (PNPG), hydroxybenzotriazole (HOBt), *N*,*N’*-diisopropylcarboiimide (DIC), acrylic acid *N*-hydroxysuccinimide (Ac–NHS ester), deuterium oxide (D_2_O), dimethylsulfoxide-*d_6_* (DMSO-*d*), chloroform-d (CDCl_3_), 2,2-dihydroxyindane-1,3-dione (Ninhydrin) and tris(hydroxymethyl)aminomethane hydrochloride (Tris–HCl) was purchased from Sigma Aldrich (St. Louis, MO, USA). GGRSK was custom-synthesized from Lifetein (Somerset, NJ, USA) and Cosmogenetech (Seoul, South Korea). Urokinase (EC 3.4.4.21)-type plasminogen activator (uPA) was purchased from Fortunachem (Wuhan, China). Acrylic acid (AAc), sodium hydroxide (NaOH), ethyl acetate anhydrous (EA), dimethylformamide (DMF), tetrahydrofuran (THF), chloroform (CCl_4_), methanol (MeOH), *n*-butanol (BuOH), acetic acid, acetonitrile, and trifluoroacetic acid were purchased from Daejung (South Korea). 2-hydroxy-2-methlypropiophenone (Darocur 1173) and dimethylsulfoxide anhydrous (DMSO) were purchased from TCI (Tokyo, Japan).

### 2.1. Synthesis of N-Carboxyanhydride (NCA) Monomer

NCA was used as received or synthesized using either of the two methods [13,14]. Briefly, *N*-boc glycine, triphosgene, and triethylamine at a molar ratio of 2.5:1.0:2.7 were dissolved in anhydrous ethyl acetate at room temperature. The reaction bath was connected to a manometer to monitor CO_2_ evolution while vigorously stirring the reaction suspension for 3 h. The solid triethylammonium hydrochloride salt was washed with ethyl acetate, and the filtrate was dried by evaporation. The resulting powder was recrystallized from chloroform and ether at −20 °C to give white crystals. Alternatively, anhydrous ethyl acetate containing glycine, *σ*-pinene at a molar ratio of 0.26:1.0 was stirred under reflux in an oil bath maintained at 90 °C. After 30 min, a 0.5× molar amount of triphosgene was added to the solution, and the reaction proceeded for 3 h. The volume of the solution was reduced to ~70% using a rotary evaporator. The product was precipitated by dropping in *n*-heptane. After recrystallization, white crystals were obtained.

### 2.2. Polymerization of NCA Using Allylamine

NCA was polymerized by a ring-opening polymerization using a primary amine as an initiator [15,16]. Briefly, 10 mmol of NCA was dissolved in DMF and 0.07 mmol of allylamine (or 2-AMEA) was added. The reaction was stirred at room temperature for 4 days under a dry nitrogen atmosphere. The solution was precipitated in ethyl acetate, filtered, and dried in a vacuum. The chemical structure was confirmed using the ^1^H-NMR spectroscopy (Varian Unity Inova 500, Varian, Palo Alto, CA, USA) (Figure S1 in supplementary material).

### 2.3. Synthesis of Vinyl-Attached PNPG

PNPG tethered with a vinyl group was synthesized as follows. Briefly, 0.1 mmol of PNPG and 2x molar amounts of HOBt and DIC were dissolved in DMF maintained at 0 °C. After 30 min, 2x and 4x molar amounts of allylamine and triethylamine were added, and the reaction proceeded overnight at room temperature. The extent of reaction was examined using TLC (a mobile phase CCl_4_: MeOH = 6:4, *R*_f_ of product = 0.78) (Figure S2 in supplementary material). The mixture was poured into hexane, stored in a freezer overnight, filtered to remove DCU, washed successively with ethyl acetate, 4% HCl, saturated NaHCO_3_ solution, and brine, dried with anhydrous MgSO_4_, and finally lyophilized. The chemical structure of the product was confirmed using the ^1^H-NMR and FT–IR spectroscopy (Nicrolet 380, Thermo Scientific, Waltham, MA, USA) (Figure S2 in supplementary material).

### 2.4. Synthesis of a Crosslinker Using the uPA Substrate

The end modification of the uPA substrate, GGRSK peptide with acrylamide (Ac–GGRSK–Ac) proceeded as follows. Briefly, the GGRSK peptide and Ac–NHS ester at a molar ratio of 1:2 were dissolved in anhydrous DMSO containing 30 μL of triethylamine. The reaction mixture was stirred overnight at room temperature. The extent of the reaction was examined using TLC (a mobile phase BuOH: Acetic acid: D_2_O = 3:1:1 with ninhydrin, R_f_ of product = 0.63) (Figure S4 in supplementary material). After concentrating the reaction mixture by evaporating DMSO using a centrifugal evaporator for 12 h at room temperature, the mixture was precipitated in THF and EA respectively to obtain Ac–GGRSK–Ac (yield: 92%). To remove low molecular weight impurities, the product was purified using a desalting spin column (89870, Thermo Fisher Scientific, Waltham, MA, USA, 50% acetonitrile solution with 0.1% trifluoroacetic acid). The chemical structure of Ac–GGRSK–Ac was confirmed using the ^1^H-NMR, FT–IR spectroscopy (Figure S3 in supplementary material), and ESI-MS (Varian 500-Ms, Agilent, Santa Clara, CA, USA). 

### 2.5. Characterization of LAP Substrates Degradation

The degradation of the LAP substrates was quantified using UV–Vis spectroscopy [17]. Briefly, 2.5 μL of the LAP was added to 500 μL of Tris–HCl buffer (50 mM, pH 7.6) containing 7 mM of triglycine or end-modified oligo (glycine). The mixture was incubated at 37 °C for a varying amount of time after which the mixture was placed in an ice bath to practically cease enzymatic activity. For the colorimetric assay [18], the mixture was diluted with 3 mL of Tris-buffer, and 5 μL of 5% *w*/*v* ninhydrin in ethanol was added to the mixture. After 5 min, the absorbance at 566 nm was measured using a UV–Vis spectrometer (Cary 100 UV–Vis, Agilent Tech., Santa Clara, CA, USA).

### 2.6. Characterization of GLU Substrates Degradation

The degradation of GLU substrates was quantified using UV–Vis spectroscopy [19]. Briefly, 25 μL of GLU was added to 250 μL of Dulbecco’s PBS (pH 7.4) containing 1 mM of PNPG or PNPG tethered to a vinyl group. The mixture was incubated at 37 °C for a varying amount of time, after which 2.5 mL of 0.2 M Na_2_CO_3_ solution was added to the mixture to inhibit enzymatic activity. The degradation of the GLU substrates was quantified by measuring the absorbance of *p*-nitrophenol (400 nm) using a UV–Vis spectrometer.

### 2.7. Characterization of uPA Substrates Degradation

The degradation of uPA substrates was examined by measuring the fold-increase in the volume of SAP (*V*_fold_) immersed in the aqueous solution containing uPA [20]. Briefly, SAP was created by irradiating UV (365 nm, 10 s, or 30 s) to the aqueous pregel solution containing acrylic acid, Ac–GGRSK–Ac, NaOH, and photo-initiator (Darocur 1173) at a molar ratio indicated in Table S2 (in supplementary material). The resulting SAP was thoroughly washed with water and immersed in the aqueous solution containing 1 μM of uPA. After incubation at 37 °C for a varying amount of time, the SAP was washed with Dulbecco’s PBS (pH 7.4) multiple times to cease the enzymatic cleavage reaction. The biodegradability of SAP was determined by measuring *V*_fold_.

## 3. Results and Discussion

### 3.1. Degradation Characterization of the End-Modified LAP Substrate

**Figure 2:**
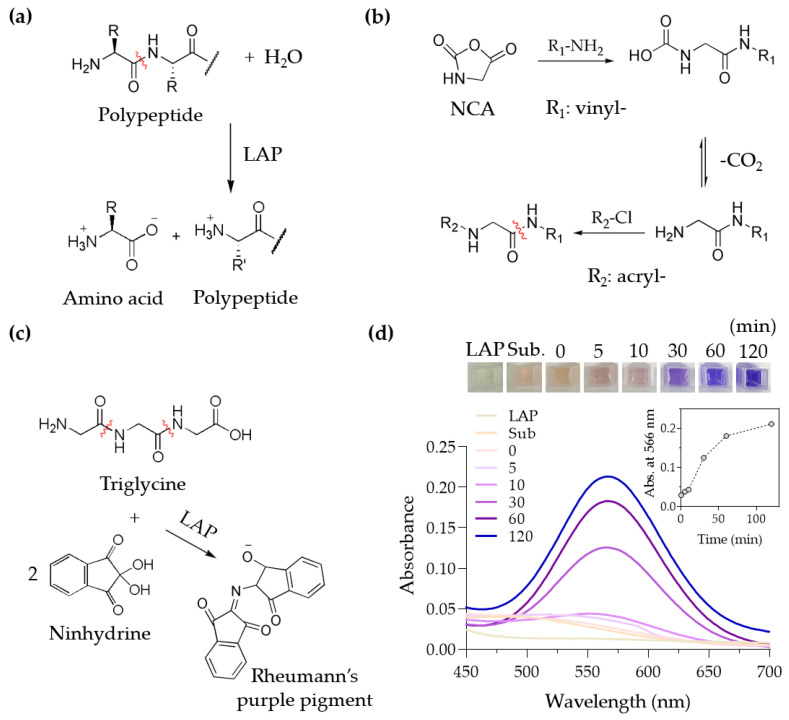
Synthesis of the end-modified LAP substrate and its biodegradability. (**a**) LAP catalyzes the hydrolysis of the amide bond at the *N*-terminus of a peptide. (**b**) Ring-opening polymerization of NCA is initiated by allylamine, and the *N*-terminus of the oligo (glycine) was modified with acryloyl chloride to become acrylamide. (**c**) When triglycine is cleaved by LAP, a primary amine is generated and reacts with ninhydrin to produce Rheumann’s purple pigment, which can be quantitatively measured using a spectroscopic method. (**d**) A photograph of purple pigments produced as a result of the triglycine cleavage by LAP at a varying incubation time (**top**) and the UV–Vis spectra (**bottom**) (inset: absorbance at 566 nm).

Of the substrates of the urinary enzymes to be modified into a cleavable crosslinker, we first examined LAP, the aminopeptidase that cleaves the amide bond to release an amino acid at the *N*-terminus (Figure 2a) [21]. Although LAP is most efficient at releasing a leucine located at the *N*-terminus, as the name indicates, LAP is considered to have broad specificity; other amino acids such as glycine can also be released by LAP [22]. We made a peptide composed only of water-soluble glycine and modified the peptide’s ends with polymerizable groups to create a crosslinker for SAP. To create an oligo (glycine), we performed the ring-opening polymerization of *N*-carboxyanhydride (NCA) using a primary amine as an initiator [16]. We used allylamine as an initiator to allow the one end of oligo (glycine) to possess a polymerizable vinyl group. The other end, a primary amine, can further be converted into a polymerizable acrylamide group by reacting with acryloyl chloride (Figure 2b). The chemical structure of the oligo (glycine) modified with polymerizable groups was confirmed by ^1^H-NMR (Figure S1).

The degradation of unmodified oligo (glycine) by LAP was examined by a triglycine. When LAP degrades triglycine, one end of the triglycine is converted to a primary amine, which can be spectroscopically detected at 566 nm by reacting with ninhydrin (Figure 2c). Using UV–Vis spectroscopy, it was confirmed that unmodified triglycine was degraded by LAP over time (Figure 2d). However, we found that the synthesized oligo (glycine) end-modified with polymerizable groups was rarely degraded by LAP, which is in accordance with the previous studies that peptides with the α-amino group substitution, for example by acetylation, were not as effectively cleaved by LAP or other aminopeptidases [23,24]. Taken together, the biodegradability of the LAP substrate was compromised by the end-modification, preventing its use as a cleavable crosslinker.

### 3.2. Degradation Characterization of the End-Modified GLU Substrate

**Figure 3:**
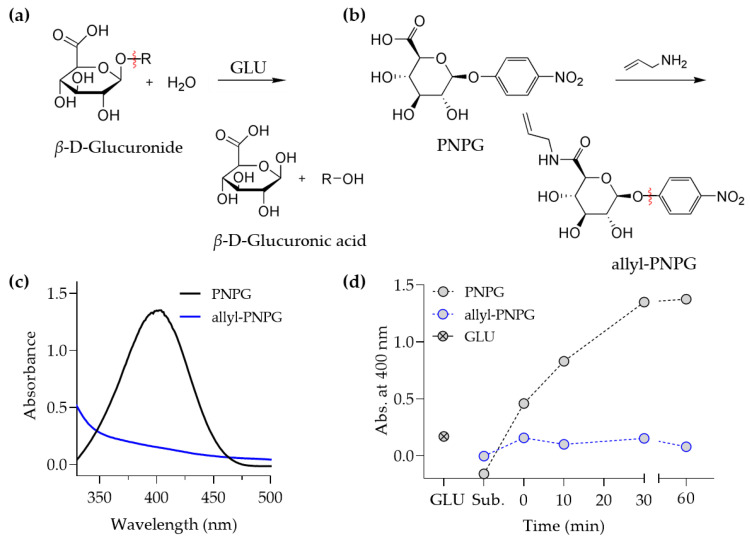
Synthesis of the allyl-substituted GLU substrate and its biodegradability. (**a**) GLU, a member of the glycosidase family, catalyzes the cleavage of a glycosidic bond at the non-reducing end and releases *β-*D*-*glucuronic acid from polysaccharides. (**b**) A modification of a carboxylic acid of PNPG with allylamine to tether the polymerizable allyl group. (**c**) UV–Vis spectra of PNPG and PNPG tethered with the allyl group (allyl-PNPG) after mixing with GLU for 30 min. PNPG displayed the characteristic spectrum of *p*-nitrophenol. (**d**) The absorbance was measured at 400 nm for PNPG and allyl-PNPG mixed with GLU over a varying incubation time.

We next examined the substrate of GLU for its potential usage as a biodegradable crosslinker for SAP. A urinary enzyme, GLU is known to catalyze the cleavage of a glycosidic bond located at the non-reducing end of carbohydrates such as oligo (*β*-D-glucuronide) [25] (Figure 3a). We then tested to see if modifying a substituent of a six-membered ring with a polymerizable group affected the degradability of the glycosidic bond by GLU. A carboxylic acid of 4-nitrophenyl-*β-*D*-*glucuronide (PNPG), a spectroscopically detectable substrate of GLU, was reacted with allylamine to tether a polymerizable vinyl group to PNPG (Figure 3b). The chemical structure of the PNPG modified with allylamine was confirmed by ^1^H–NMR, FT–IR, and thin-layer chromatography (TLC) (Figure S2). The degradability of PNPG by GLU can be examined by measuring the absorbance of *p*-nitrophenol, which can be released from PNPG by GLU via cleavage of a glycosidic bond. When GLU was added to an aqueous solution containing PNPG, a characteristic absorbance spectrum of *p*-nitrophenol with a peak wavelength at 400 nm was observed (Figure 3c), which indicated that PNPG was degraded by GLU. When GLU was added to an aqueous solution containing PNPG tethered with an allyl group, however, the characteristic absorbance spectrum of *p*-nitrophenol was not observed (Figure 3c). Throughout incubation time, the absorbance at 400 nm remained at a basal level for PNPG tethered with allyl group, whereas unmodified PNPG showed a steady increase in absorbance at 400 nm (Figure 3d). Therefore, we concluded that the biodegradability of the GLU substrate was compromised by the vinyl group attachment, and the oligo (*β-*D*-*glucuronide) modified with the vinyl group cannot be used as the biodegradable crosslinker for SAP.

### 3.3. The End-Modified uPA Substrate

**Figure 4:**
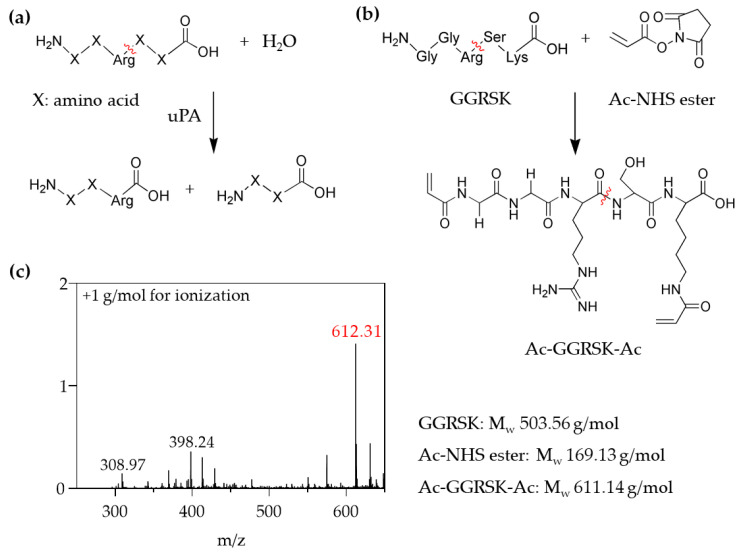
Synthesis of Ac–GGRSK–Ac to be used as the biodegradable crosslinker. (**a**) uPA cleaves the *C*-terminus of Arg in the peptide sequence. (**b**) The *N*-terminus of GGRSK and the primary amine of the lysine sidechain react with Ac–NHS ester to create Ac–GGRSK–Ac. (**c**) A mass spectrum of Ac–GGRSK–Ac after purification.

Confirming that the chemical modification of the LAP and GLU substrates with polymerizable groups prevented their biodegradation by the corresponding enzymes, we examined the uPA substrate for its biodegradability when modified with a polymerizable group. The uPA substrate is a trypsin-like protease that catalyzes plasminogen to plasmin in plasma by cleaving the peptide bond formed between the *C*-terminus of an arginine (Arg) residue and the *N*-terminus of adjacent amino acid residues (Figure 4a). Previous studies used the commercial substrates of uPA, where the *C*-terminus of Arg was substituted with *p*-nitroanilide, 4-methylcoumarin, or fluorescent dyes, which are spectroscopically detectable when released by uPA [26,27,28]. Peptides with various sequences containing Arg were examined for cleavage by uPA. For example, the X_2_–X_1_–Arg↓X_1_^′^substrate containing the fluorogenic leaving group (X_1_^′^), where X_1_ (Gly, Ala or Ser) and X_2_ (Thr or Ser) are connected to the *N*-terminus of Arg, was effectively degraded by uPA at the *C*-terminus of Arg [26]. Besides, the peptide Gly–Arg↓X, the native sequence derived from plasminogen, was more efficiently cleaved by uPA when X was Ser than Var [29].

Based on the previous results, we choose the peptide sequence Gly–Gly–Arg–Ser–Lys (GGRSK) for the chemical modification in the biodegradable crosslinker. The *N*-terminus of glycine and *ε*-amino group of lysine reacted with acrylic acid *N*-hydroxysuccinimide ester (Ac–NHS ester) to create polymerizable acrylamide groups at both ends (Ac–GGRSK–Ac) (Figure 4b). We expected that two the glycine residues inserted between the *N*-terminus and Arg as well as a long alkyl side chain of lysine would provide sufficient distance between the polymerizable groups and the cleavage site (Arg–Ser). Thus, we anticipated that uPA can bind to the cleavage site without steric hindrance by the acrylamide groups and subsequent PAA chains upon polymerization into SAP. The chemical structure of the modified peptide was confirmed using FT–IR and ^1^H–NMR spectroscopy (Figure S3); besides, we performed the electrospray ionization mass spectroscopy (ESI–MS) to measure the molecular weight of the modified peptide Ac–GGRSK–Ac. The molecular weight measured at a maximum intensity of 612.3 g/mol exactly matched the value calculated for Ac–GGRSK–Ac (+1 g/mol for ionization) (Figure 4c). TLC with ninhydrin treatment further confirmed the crosslinker formation (Figure S4 in supplementary material).

### 3.4. Degradation Characterization of the SAP Crosslinked by the uPA Substrate

**Figure 5:**
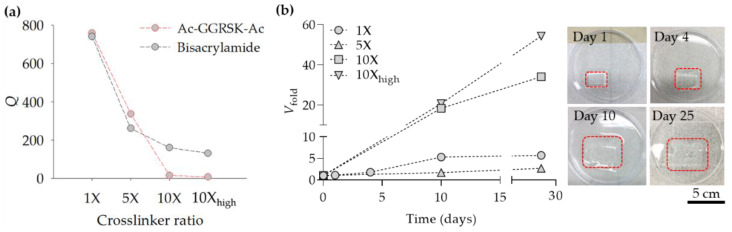
*Q* and *V*_fold_ measure for the SAP created with Ac–GGRSK–Ac. (**a**) *Q* measured for the SAP created with the degradable and non-degradable crosslinkers at a varying crosslinker molar ratio and UV irradiation dose. (**b**) *V*_fold_ measured over the incubation time for the SAP swollen in PBS containing 1 μM uPA. In the images, the boundary of SAP is indicated by a dashed red line. The scale bar is 5 cm.

Using Ac–GGRSK–Ac, we created PAA-based SAP and measured its equilibrium swelling ratio *Q*, which is defined as the weight ratio of SAP at an equilibrium swelling state to a dried state. SAP was synthesized by the UV irradiation (365 nm) of the precursor solution containing acrylic acid, Ac–GGRSK–Ac, and the photo-initiator. The molar ratios between each component were similar to those commonly used in the SAP industry (Table S2), and the SAP made from the molar ratios was indicated as 1X. We also created SAPs with a 5- and 10-fold increase in the Ac–GGRSK–Ac molar ratio (compared to that of 1X SAP), which were indicated as 5X SAP and 10X SAP, respectively. *Q* measured for 1X SAP was 760 but was reduced to 336 and 15 for 5X SAP and 10X SAP, respectively (Figure 5a). When 10X SAP was created with a high dose of UV irradiation to increase the conversion of Ac–GGRSK–Ac, which we indicated as 10X_high_ SAP, *Q* became substantially lower. We also measured the *Q* of the SAP made of the non-degradable crosslinker (bisacrylamide) and compared the results with those obtained using the degradable SAP. We found that *Q* was sufficiently similar between the degradable and non-degradable SAPs when the molar ratio of the crosslinker was 1X and 5X, indicating that the absorption ability of the SAP was not compromised by biodegradability. When the molar ratio of the crosslinker was higher (10X and 10X_high_), *Q* became significantly lower for the degradable SAP than the non-degradable SAP. We noted that the degradable crosslinker (*M*_w_ = 612 g/mol) was longer, and thus, more flexible than the non-degradable one (*M*_w_ = 154 g/mol). Therefore, when the crosslinker was tethered to the polymer network at the one end (acrylamide), the reaction of the other end (acrylamide) with a growing polymer chain was more efficient for the longer degradable crosslinker especially when the crosslinking density was high.

Next, we examined the SAP made of Ac–GGRSK–Ac for its biodegradability by uPA. SAP made of Ac–GGRSK–Ac and swollen in an equilibrium state was incubated in the phosphate buffered saline (PBS, 37 °C) containing uPA at 1 μM, which is similar in concentration to that found in human urine (0.3 μM) [30]. We found that the volume of SAP swollen in PBS, denoted as the fold-increase in volume *V*_fold_ increased with incubation time (Figure 5b). The SAP made with a higher molar ratio of crosslinker and irradiated with a higher UV dose showed a steeper increase of *V*_fold_ with incubation time, indicating that the crosslinker was more rapidly cleaved by uPA. After 30 days, the SAP swelled so much it became practically shapeless, indicating that the crosslinker was mostly cleaved by uPA. We infered that the time required for complete degradation can be matched to the times necessary for individual applications by adjusting the crosslinker density and the degradation kinetics of each crosslinker. In practice, the temperature and the quality of aqueous solution would affect *Q* [31,32], which in turn would influence the crosslinker density and degradation kinetics. Taken together, we demonstrated the creation of the biodegradable SAP using the cleavable crosslinker made of the GGRSK peptide, the substrate of uPA. As a control experiment, we created the SAP using a non-cleavable crosslinker, bisacrylamide, and found that *V*_fold_ was maintained at 1 over an extended incubation time.

## 4. Conclusions

In summary, we created a biodegradable SAP using a crosslinker cleavable by the urinary enzyme uPA. We chose the substrates of three human urinary hydrolases (LAP, GLU, and uPA) to examine whether the substrates were enzymatically cleaved when modified at both ends by polymerizable groups. We found that the LAP and GLU substrates, oligo (glycine) and oligo (*β*-D-glucuronide), respectively, that had been end-modified with polymerizable groups were rarely cleaved by their respective enzymes, presumably due to steric hindrance provided by the polymerizable groups. When the uPA substrate, GGRSK, was end-modified with acrylamide groups (Ac–GGRSK–Ac), however, we found that Ac–GGRSK–Ac was successfully degraded by uPA in a physiological concentration (1 μM) as demonstrated by a continuous increase of *V*_fold_ of the SAP made of Ac–GGRSK–Ac. We suggest that the sufficient distances between the polymerizable groups and the enzymatic cleavage site were the critical factor for determining biodegradability. This was the first example of using a substrate of the human urinary enzyme, uPA, to create biodegradable PAA-based SAP, which can potentially be used in the diaper industry. However, due to the high cost of GGRSK–peptide synthesis, the peptide crosslinker in its present form cannot be used for the industry-scale production. Therefore, future studies should aim at finding inexpensive, mass-producible peptides or proteins that contain the uPA cleavable sequence. Alternatively, a method of immobilizing other microbial proteases inside the SAP, if they are safe for use, and releasing them upon the urine absorption would widen the choice of a biodegradable crosslinker. Moreover, this method would result in more reliable degradation of the SAP regardless of urine composition, which may fluctuate depending on age, medical condition and levels of dilution. Future studies should also aim to embed hydrolytically cleavable sites, such as ester groups, into the backbone of linear PAA chains. In that case, high-molecular weight PAA chains degraded from SAP by uPA can further be fragmented into PAA chains of the molecular weight smaller than 1 kg/mol, which can then be degraded by soil microorganisms found in sewage. Cyclic ketene acetal, such as 2-methylene-1,3-dioxepane, would be one ester group candidate for introduction into the backbone of PAA when radically copolymerized by acrylic acid [33].

## Data Availability

Data is contained within the article or supplementary material.

## Supplementary Materials

The following are available online at https://www.mdpi.com/2073-4360/13/6/929/s1, Figure S1: ^1^H-NMR spectrum of NCA polymerization initiated by primary amine, Figure S2: Synthesis of PNPG compound, Figure S3: Synthesis of peptide crosslinker, Figure S4: TLC of Ac–GGRSK–Ac, Table S1: Detailed information on enzymes used in the study, Table S2: The pregel composition used to create SAP.
