# Supplementary material for: Superabsorbent Polymer Network Degradable by a Human Urinary Enzyme
Source: Polymers (Basel). 2021 Mar 17;13(6):929. doi: 10.3390/polym13060929 (PMC8002648; doi:10.3390/polym13060929)
Supplement: Supplementary file 1 [file polymers-13-00929-s001.pdf]

Supplementary information for

Superabsorbent polymer network degradable by a human urinary enzyme

Minji Whang [1], Hyeonji Yu [1] and Jungwook Kim *

[1]Department of Chemical and Biomolecular Engineering, Sogang University, 35,Baekbeom-ro, Mapo-gu, Seoul, Republic of Korea 04107; mjwhang@sogang.ac.kr (M.W.); lkjh6309@sogang.ac.kr (H.Y.)

*Correspondence: jungwkim@sogang.ac.kr; Tel.: +82-2-704-8793

Contents

Supplementary Figure 1 to 4

Supplementary Table 1 to 2

References

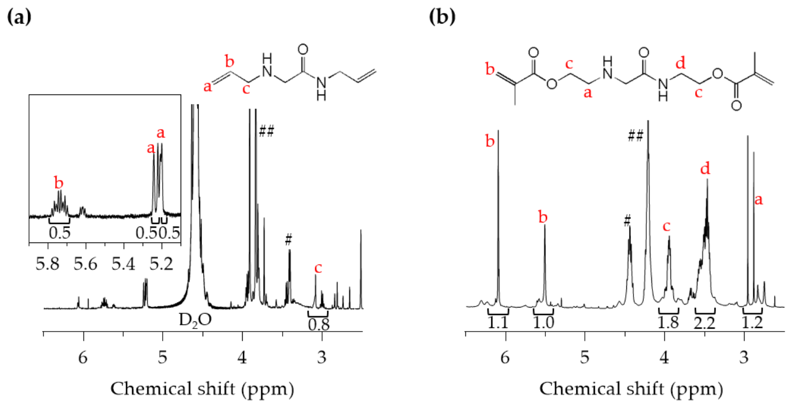

Figure S1. ^{1}H-NMR spectrum of NCA polymerization initiated by primary amine. (**a**) Resulting from the reaction of allyl amine. *a*, *b* indicates CH(*m*), CH_2(*m*) of vinyl group at 5.2~5.3, 5.6~5.8 ppm and *c* indicates CH_2(*t*) at 2.9~3.1 ppm. #, ## indicates residual allylamine, 2,5-oxazolidinedione at 3.4, 3.8 ppm. The residual D_2O peak appears at 4.8 ppm. (**b**) Resulting from the reaction of 2-aminoethyl methacrylate. *a*, *b*, *c* indicates CH_2(*q*), CH(*m*), CH_2 (*t*) at 2.8~3.0, 5.5~6.1 of methacrylate group, 3.9 ppm and d indicates CH_2(*q*) at 3.5 ppm. #, ## indicates residual 2-AMEA at 4.2, 4.5 ppm.

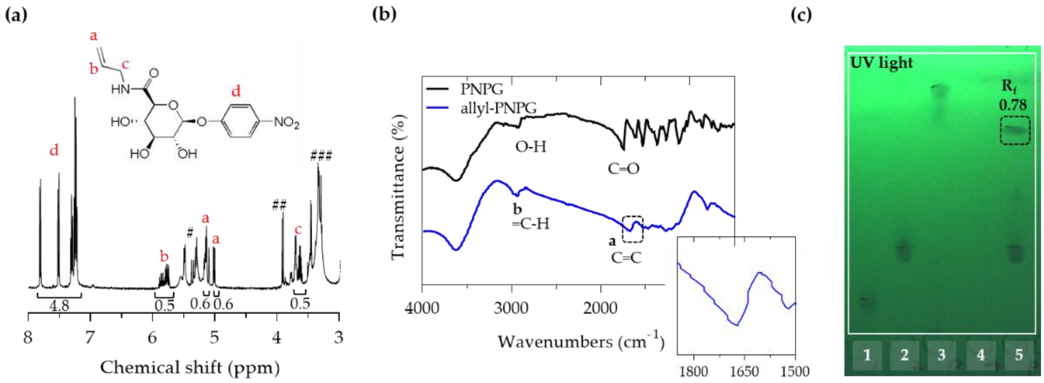

Figure S2. Synthesis of PNPG compound. (**a**) ^{1}H-NMR spectrum of allyl-PNPG. *a*, *b* indicates CH(*t*), CH(*m*) of vinyl group at 5.0~5.1, 5.6~5.9 ppm. *c*, *d* indicates CH_2(*t*), CH(*m*) at 3.5~3.7, .2~7.8 ppm. # indicates residual allylamine at 5.5 ppm. ##, ### indicates residual PNPG at 3.5, 3.9 ppm. (**b**) FT-IR spectrum of PNPG and allyl-PNPG. *a* indicates C=C bond at 1640~1680cm^{-1} (inset), *b* indicates =C-H bond at 3000~3100 cm^{-1}. (**c**) TLC of allyl-PNPG. (Marked: (1) PNPG, (2) HOBt, (3) DIC, (4) allylamine, (5) allyl-PNPG (R_f: 0.78))

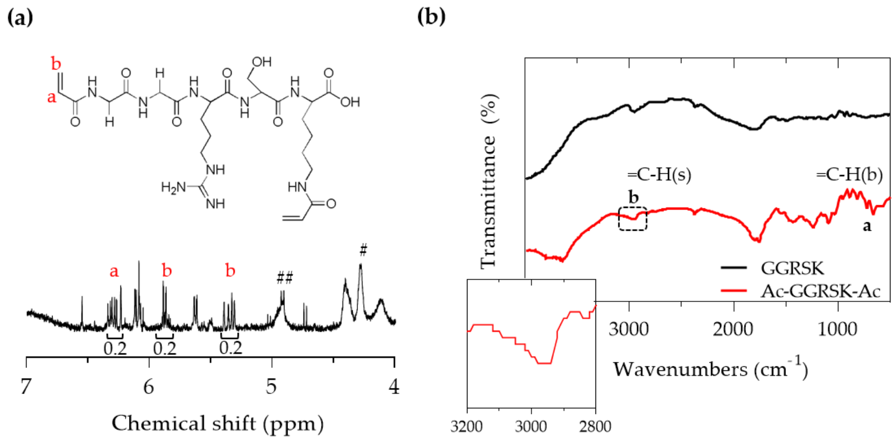

Figure S3. Synthesis of peptide crosslinker. (**a**) ^{1}H-NMR spectrum of Ac-GGRSK-Ac. *a*, *b* indicates CH(*m*), CH(*t*) of acrylamide at 6.2~6.5, 5.8~5.9 ppm. #, # indicates residual GGRSK at 4.0~4.5, 4.8 ppm. (**b**) FT-IR spectrum of GGRSK and Ac-GGRSK-Ac. *a* indicates =C-H(*b*) group at 650~1000 cm^{-1} and *b* indicates =C-H(*s*) group at 3000~3100 cm^{-1} (inset).

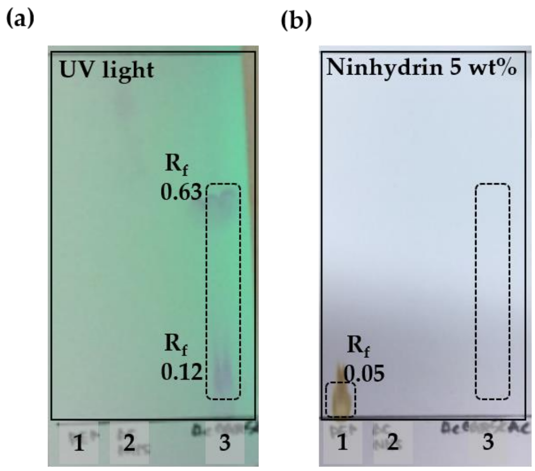

Figure S4. TLC of Ac-GGRSK-Ac. (**a**) UV light. (**b**) 5 wt% Ninhydrin. (Marked: (1) GGRSK, (2) Ac-NHS ester, (3) Ac-GGRSK-Ac (R_f: 0.63))

Table S1. Detailed information on enzymes used in the study.

	Full name	EC #	Molecular weight (kDa)	Optimal pH	Isoelec. point	Specificity	Unit (U) /urine (mL)
**LAP**	*Leucine aminopeptidase*	3.4.11.1	~ 326	9.0 - 9.5	6.07	Release amino acids from the N-terminus of proteins. React efficiently with leucine.	Male: 82<br>Female: 43 [1]
**GLU**	*β-D-glucuronidase*	3.2.1.31	~ 290	4.5 - 5.0	4.8	Catalyze the conversion of *β*-D-glucuronoside to D-glucuronate	8.8 - 28.4 [2]
**uPA**	*Urokinase-type plasminogen activator*	3.4.21.73	33 ~ 54	8.5	8.78	A serine protease that cleaves plasminogen to create plasmin.	4.1 [3]

Table S2. The pregel composition used to create SAP.

	Chemicals	Molar ratio (mol %)
Monomer	Acrylic acid	100
Crosslinker (1X)	Ac-GGRSK-Ac	0.041
Photo initiator	Darocur 1173	0.065
Solvent	NaOH	70
	Water	523

References

1. Goldbarg, J. A.; Rutenburg, A. M., The colorimetric determination of leucine aminopeptidase in urine and serum of normal subjects and patients with cancer and other diseases. *Cancer* **1958,** *11* (2), 283-91.
2. Wu, X.; Loganathan, D.; Linhardt, R. J., Sensitive method for the quantification of beta-glucuronidase activity in human urine using capillary electrophoresis with fluorescence detection. *J Chromatogr B Biomed Sci Appl* **1998,** *708* (1-2), 61-6.
3. Hong, S. Y.; Yang, D. H.; Lee, B. H.; Ki, E. K.; Chung, K. H., The urine urokinase concentration in end stage renal disease with acquired renal cyst. *Korean J Intern Med* **1991,** *6* (2), 64-8.
